# Impact of the coronavirus disease 2019 (COVID-19) pandemic on nosocomial *Clostridioides difficile* infection

**DOI:** 10.1017/ice.2020.454

**Published:** 2020-09-08

**Authors:** Manuel Ponce-Alonso, Javier Sáez de la Fuente, Angela Rincón-Carlavilla, Paloma Moreno-Nunez, Laura Martínez-García, Rosa Escudero-Sánchez, Rosario Pintor, Sergio García-Fernández, Javier Cobo

**Affiliations:** 1 Servicio de Microbiología, Hospital Universitario Ramón y Cajal and Instituto Ramón y Cajal de Investigación Sanitaria, Madrid, Spain; 2 Red Española de Investigación en Patología Infecciosa, Madrid, Spain; 3 Servicio de Farmacia Hospitalaria, Hospital Universitario Ramón y Cajal and Instituto Ramón y Cajal de Investigación Sanitaria, Madrid, Spain; 4 Servicio de Medicina Preventiva, Hospital Universitario Ramón y Cajal and Instituto Ramón y Cajal de Investigación Sanitaria, Madrid, Spain; 5 Servicio de Enfermedades Infecciosas, Hospital Universitario Ramón y Cajal and Instituto Ramón y Cajal de Investigación Sanitaria, Madrid, Spain

## Abstract

**Objectives:**

The coronavirus disease 2019 (COVID-19) pandemic has induced a reinforcement of infection control measures in the hospital setting. Here, we assess the impact of the COVID-19 pandemic on the incidence of nosocomial *Clostridioides difficile* infection (CDI).

**Methods:**

We retrospectively compared the incidence density (cases per 10,000 patient days) of healthcare-facility–associated (HCFA) CDI in a tertiary-care hospital in Madrid, Spain, during the maximum incidence of COVID-19 (March 11 to May 11, 2020) with the same period of the previous year (control period). We also assessed the aggregate in-hospital antibiotic use (ie, defined daily doses [DDD] per 100 occupied bed days [BD]) and incidence density (ie, movements per 1,000 patient days) of patient mobility during both periods.

**Results:**

In total, 2,337 patients with reverse transcription-polymerase chain reaction–confirmed COVID-19 were admitted to the hospital during the COVID-19 period. Also, 12 HCFA CDI cases were reported at this time (incidence density, 2.68 per 10,000 patient days), whereas 34 HCFA CDI cases were identified during the control period (incidence density, 8.54 per 10,000 patient days) (*P* = .000257). Antibiotic consumption was slightly higher during the COVID-19 period (89.73 DDD per 100 BD) than during the control period (79.16 DDD per 100 BD). The incidence density of patient movements was 587.61 per 1,000 patient days during the control period and was significantly lower during the COVID-19 period (300.86 per 1,000 patient days) (*P* < .0001).

**Conclusions:**

The observed reduction of ~70% in the incidence density of HCFA CDI in a context of no reduction in antibiotic use supports the importance of reducing nosocomial transmission by healthcare workers and asymptomatic colonized patients, reinforcing cleaning procedures and reducing patient mobility in the epidemiological control of CDI.

*Clostridioides difficile* is the leading cause of nosocomial infectious diarrhea and one of the most prevalent nosocomial pathogens.^1,2^ The key elements that determine its incidence are exposure to *C. difficile* spores and the administration of antibiotics.^3^ Controversy exists over the utility of various infection control measures, given that most interventions have shown very low levels of evidence,^4^ whereas bundle-based programs that include antibiotic restriction are almost always effective.^4,5^

The COVID-19 pandemic in Spain has been particularly intense, with >6,000 cases per million inhabitants and exceeding 28,000 deaths^6^; it was even more serious in the capital Madrid. Our hospital suddenly became a monographic COVID-19 hospital, with a peak in the pandemic on March 30, 2020, when 86.91% of admitted patients (983 of 1,131) were diagnosed with COVID-19. This situation in which almost all patients remained isolated and all healthcare workers wore personal protective equipment (PPE) constituted a type of “natural experiment” for the study of *C. difficile* epidemiology in hospitals. The objective of this study was to assess the impact of the COVID-19 pandemic on the incidence of CDI and to analyze the factors that could have influenced the incidence.

## Methods

We compared the incidence density (cases per 10,000 patient days) of nosocomial CDI in a tertiary-care teaching hospital in Madrid over 2 periods: (1) the peak incidence of COVID-19 at our hospital (COVID-19 period: March 11, 2020, to May 11, 2020) and (2) the same period of the previous year (control period: March 11, 2019, to May 11, 2019). We used the standard epidemiological classification of CDI,^7^ only considering hospital-onset healthcare facility-associated (HO-HCFA) and community-onset healthcare facility-associated (CO-HCFA) infections as nosocomial, and ruling out community, indeterminate, and recurrence cases for the incidence density calculation. We also calculated the incidence density for all types of CDI during the interval between the 2 periods to better describe the time trend of cases. For the COVID-19 period, we reviewed whether the hospitalized patients who were screened for CDI also presented a diagnosis of COVID-19, with microbiological confirmation by reverse transcription-polymerase chain reaction (RT-PCR). We used the computerized registry of the microbiology department to obtain all data regarding CDI and COVID-19 tests, and we gathered hospital admission data regarding hospital stays. The algorithm employed for the microbiological diagnosis of CDI was the same throughout the study period, which included sequential qualitative detection of glutamate dehydrogenase (C. DIFF QUIK CHEK, TechLab, Blacksburg, VA) and A and B toxins (TOX A/B QUIK CHEK, TechLab) from *C. difficile.* We assessed discrepancies by detecting the *C. difficile* toxin B gene by RT-PCR (BD MAX Cdiff, Becton Dickinson, Franklin Lakes, NJ).

We assessed patient mobility by determining all of the patients’ administrative location changes during both periods using the hospital information systems service. These changes included transfers to the operating room, change in room or change to intensive care units, as well as transfers to perform additional tests (eg, radiological examinations, endoscopies, or other procedures). We then calculated the “incidence density” of the patients’ movements by dividing the sum of the location changes by the hospital stays in both periods.

For the same study period, we extracted aggregate in-hospital antibiotic use data from the computerized hospital administration records and an automated medication-dispensing system. Data on antibiotic use are expressed as defined daily doses per 100 occupied bed days (DDD per 100 BD) and the percentage of change between study periods, according to the criteria of the World Health Organization Collaborating Centre for Drug Statistics Methodology. We also extracted data regarding time of exposure to antibiotics, expressed as days of therapy (DOT) per 100 patient days. For this study, we considered only the consumption of antibiotics from Anatomical Therapeutic Chemical group J01. Finally, as a subrogate measurement of antibiotic exposure, the proportion of admitted patients who suffered from at least 1 episode of microbiologically confirmed nonviral infection was calculated for both periods.

We compared the categorical data using the χ^2^ test and the Student *t* test to compare the continuous variables when a normal distribution could be assumed or the Mann-Whitney U test otherwise. We assessed the normality of the continuous variables using the Shapiro-Wilk test.

## Results

In total, 2,337 patients with RT-PCR-confirmed COVID-19 and 283 with suspected COVID-19 were admitted to the hospital during the COVID-19 period. There were 44,831 hospital stays during this time, whereas during the same period of 2019 there were 39,795 stays. The mean age of the admitted patients was 66.74±17.65 years for the COVID-19 period and 64.41±21.23 years for the control period (*P* < .0001).

### Clostridioides difficile *infection cases*

**Fig. 1.**
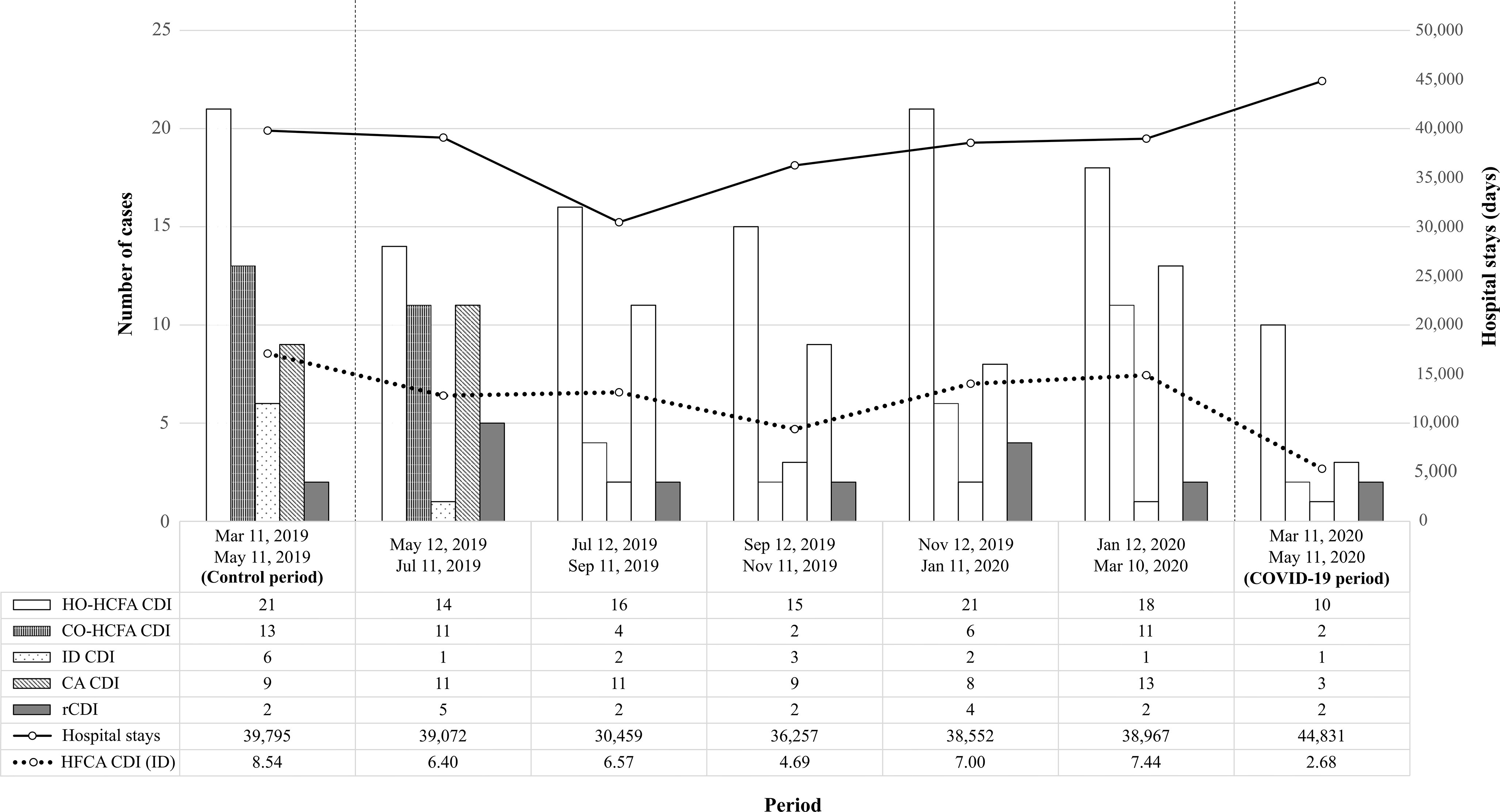
Evolution of *C. difficile* infection (CDI) over time, from control period (left) to COVID-19 period (right). The bar chart shows the total CDI case count, grouped by epidemiological definition. The solid line represents total hospital stays during each period (in days), which were used to calculate the incidence density of nosocomial CDI cases (dashed line). Note. HO-HCFA CDI, hospital-onset healthcare facility-associated *C. difficile* infection; CO-HCFA CDI, community-onset healthcare facility-associated *C. difficile* infection; ID CDI, indeterminate-onset *C. difficile* infection; CA CDI, community-acquired *C. difficile* infection; rCDI, recurrent *C. difficile* infection; HCFA CDI (ID), incidence density of healthcare facility-associated *C. difficile* infection.

The total requests for *C. difficile* detection in the hospitalized patients were similar between the COVID-19 period (n = 209) and the control period (n = 203), with a 9.8% reduction in the rate of requests during the COVID-19 period (4.6 per 1,000 hospital stays vs 5.1 per 1,000 hospital stays). Also, 12 HCFA CDI cases were identified during the COVID-19 period (10 corresponding to HO-HCFA and 2 to CO-HCFA), which resulted in an HCFA CDI incidence density of 2.68 per 10,000 patient days. In the control period, we identified 34 HCFA CDI cases (21 corresponding to HO-HCFA and 13 to CO-HCFA), which resulted in an HCFA CDI incidence density of 8.54 per 10,000 patient days. Thus, the HCFA CDI incidence density was ~3 times lower for the COVID-19 period than for the non-COVID-19 period (incidence rate ratio, 0.31; 95% CI, 0.16–0.61; *P* = .000257). Figure 1 shows the evolution of CDI cases and the incidence density of HCFA CDI during the 2 study periods and the 10-month interval between them.

Among the 209 hospitalized patients screened for CDI during the COVID-19 period, a lower proportion of CDI was observed in those with RT-PCR–confirmed COVID-19 (5 of 106; 4.7%) compared with that observed in patients without a microbiological diagnosis of COVID-19 (8 of 103; 7.8%). However, these differences were not statistically significant (*P* = .36).

### Patient mobility

Patient mobility was drastically reduced during the COVID-19 period, with 4,858 bed movements (2,274 involving a nursing unit change), whereas 7,338 bed movements (1,877 involving a nursing unit change) were observed during the control period. The number of surgical interventions was reduced to 668 during the COVID-19 period, contrasting with the 2,227 surgeries observed during the control period. The total numbers of diagnostic or care procedures that involved patient movement were 7,963 for the COVID-19 period and 13,819 for the control period. The incidence density of movements was 300.86 per 1,000 patient days for the COVID-19 period and 587.61 per 1,000 patient days for the control period (*P* < .0001).

### Antibiotic consumption

The consumption of antibiotics measured by DDD per 100 BD was higher during the COVID-19 period (89.73 DDD per 100 BD) than during the control period (79.16 DDD per 100 BD), remaining stable during the months between the 2 periods (77.50±1.64 DDD per 100 BD). The qualitative analysis showed a dramatic increase during the COVID-19 period in the use of third-generation cephalosporins (6.34 DDD per 100 BD for the control period vs 19.39 DDD per 100 BD for the COVID-19 period) and macrolides (5.76 DDD per 100 BD vs 25.49 DDD per 100 BD, respectively). We observed the same trend in terms of time of exposure to both third-generation cephalosporins (6.64 DOT per 100 patient days for the control period vs 20.98 DOT per 100 patient days for the COVID-19 period) and macrolides (3.97 DOT per 100 patient days vs 15.97 DOT per 100 patient days, respectively). Conversely, we observed a remarkable reduction in the consumption of quinolones (8.68 DDD per 100 BD for the control period vs 4.61 DDD per 100 BD for the COVID-19 period) and inhibitor-penicillin combinations (25.7 DDD per 100 BD vs 16.60 DDD per 100 BD, respectively). Treatment duration was also reduced for quinolones (8.14 DOT per 100 patient days for the control period vs 3.98 DOT per 100 patient days for the COVID-19 period) and inhibitor-penicillin combinations (27.94 DOT per 100 patient days vs 17.95 DOT per 100 patient days, respectively). There were no relevant differences in the use or in treatment duration of carbapenems between the 2 periods (7.42 DDD per 100 BD for the control period vs 6.34 DDD per 100 BD for the COVID-19 period; 8.06 DOT per 100 patient days vs 6.93 DOT per 100 patient days, respectively). Finally, as an indirect measurement of antibiotic exposure, the proportion of admitted patients who suffered from at least 1 episode of microbiologically confirmed nonviral infection was higher during the control period (15.5%; 984 of 6,365 patients) than during the COVID-19 period (14%; 783 of 5,600 patients).

### Infection control measures

An infection prevention bundle was implemented during the COVID-19 period (Table 1). All healthcare workers wore personal protective equipment (PPE) when caring for patients with COVID-19 and wore nonwaterproof masks, gloves, and gowns when treating patients without COVID-19. Environmental cleaning by trained cleaning staff was reinforced, and visits were prohibited (except in exceptional situations). Patients with COVID-19 were grouped in wards, with rooms intended for a maximum of 2 patients; however, a third bed had to be included in some cases. Patients with CDI were isolated in a single room with contact precautions during both the COVID-19 and control periods. Moreover, the same cleaning products were employed for both periods.

**Table 1.** Implemented Bundle to Prevent the Spread of SARS-CoV-2 in our Hospital During the COVID-19 Pandemic

Bundle Element	Description
Personal protective equipment (PPE)	All healthcare workers wore PPE (masks, gloves, goggles, caps and waterproof gowns) to care for patients with COVID-19
	Training healthcare workers on the proper use of PPE
Patient location	Individual rooms/grouping of cases
Isolation precautions	Design and diffusion of specific posters
	Isolation precautions for almost all hospitalized patients
	Isolation of a confirmed/suspected patient in <24 h through an *ad hoc* alert system
	Isolation measures placed very visibly in the patient’s medical chart
	Increased availability of infection control staff for information and incident resolution
Patient environment (rooms, common areas and transit areas)	Reinforcement of the daily communication between infection control staff, cleaning staff and management team
	Reinforcement of the cleaning staff in all hospitalization areas
	Operation check of chlorinated product dispensing pumps to ensure adequate concentrations (3,000 ppm)
	Training reinforcement for cleaning staff
	Adaptation of the usual cleaning procedures and creation of specific protocols for SARS-CoV-2 eradication
	Design of a poster with the most relevant points of each cleaning procedure and placement in visible areas
	Acquisition of additional cleaning material to prevent its reuse
	Daily audit of scheduled cleaning by the preventive medicine department
	Schedule of special cleaning adapted to the needs detected in the audits
	Reinforcement of the cleaning of common areas with sodium hypochlorite sprays by the military emergency unit
Sanitary material	Training healthcare workers on disinfection procedures of sanitary material
	Increased availability of disinfectant products
	Audit of the proper use of cleaning products and disinfection procedures
Health worker environment	Training healthcare workers on the need to disinfect counters, computer equipment, and personal items after use
	Cleaning of the common and rest areas of health personnel after each work shift
Patient movements	Transfers limited to what is strictly essential (diagnostic or therapeutic procedures)
Visits	Extension of the prohibition of visits and companions to almost all situations
Waste management	Reduction of waste movement by installing class III waste containers inside all rooms
Hand hygiene	Training reinforcement on hand hygiene practices

## Discussion

Our results show a remarkable reduction in the incidence density of nosocomial CDI during the period with the maximum incidence of COVID-19 compared with the same period the previous year. The number of CDI cases remained stable in the previous months; thus, such a decrease during the COVID-19 period cannot be explained by the previous trend. Moreover, the number of CDI test requests for the hospitalized patients from both periods was similar; therefore, there is no reason to envision a reduction in the clinical suspicion of CDI in patients with diarrhea (despite the frequent use of lopinavir-ritonavir as COVID-19 therapy).

Based on our findings, the consumption of antibiotics does not appear to explain the decrease in CDI. Although the use of quinolones was reduced, the overall consumption of antibiotics increased during the COVID-19 period. This observation contrasts with the lower proportion of admitted patients who suffered from at least 1 episode of microbiologically confirmed nonviral infection during the COVID-19 period (14%), compared to the control period (15.5%). The increment in antibiotic consumption could be explained by the fact that, for a time, the institutional protocol for the treatment of patients with COVID-19 contemplated the optional use of empiric ceftriaxone, and many doctors added it as standard regimen from the emergency room. In fact, a recent systematic review^8^ pointed out that 71.9% of patients with COVID-19 received antibiotics despite the low rate of bacterial infection observed in those patients (6.9%). This finding could explain the noticeable increase in both the use and time of exposure to third-generation cephalosporins observed in our study, a subgroup of antimicrobial agents associated with a higher risk for developing CDI^9^.

Due to the exceptional epidemiological situation during the COVID-19 period, our institution introduced an extraordinary reinforcement of all infection control measures, including patient isolation, universal PPE, limited patient visits and movement, and reinforcement of cleaning regimens, all of which have indirectly limited the nosocomial spread of *C. difficile*. During this period, we observed an almost 70% reduction in the incidence density of nosocomial CDI. We postulate that this observation confirms the importance of strategies aimed at reducing nosocomial transmission of *C. difficile*. Notably, not only the reinforcement of infection control measures but also the exceptionally dramatic situation during the COVID-19 period could have contributed to an increase in adherence to those measures by healthcare workers, as has already been pointed out.^10,11^

The extension of containment measures to all of our hospitalized patients during the COVID-19 pandemic could have also limited transmission from asymptomatic patients, who represent an important source of transmission,^12,13^ despite this group transmitting less effectively.^14^ In addition, the suppression of consultations and surgical procedures in the hospital has meant fewer opportunities for introducing *C. difficile* into the hospital from the community.

Our results contrast with those of a recent study that found no benefits on the incidence of CDI from improving hospital cleaning procedures.^15^ Closing hospitals and transferring patients to individual rooms located in new facilities has not conclusively been associated with a reduction in CDI rates^16,17^; however, our results support previous studies that linked the mobility of patients to common areas with increased risk of developing CDI^9^ and the potential risk of transmission by the hands of healthcare workers.^18,19^ Notably, in both cases, transmission was not associated only with direct contact with symptomatic patients^20^ and multidisciplinary measures were necessary to limit the spread of *C. difficile*.

The retrospective nature of our study precludes us from controlling for numerous factors and from measuring certain variables in detail, such as the degree of compliance with cleaning and the previous state of *C. difficile* colonization of patients, which might have been lower than usual upon admission. We also cannot rule out the possibility that our COVID-19 population was mostly composed of previously healthy patients, although our data showed that patients admitted during the COVID-19 period were significantly older than those admitted during the control period. Another limitation of our study lies in the effect of the COVID-19 pandemic on outpatient care, as recently highlighted in other more serious diseases.^21^ This factor could explain part of the reduction observed in nosocomial CDI cases by reducing opportunities for CO-HCFA diagnosis and requests for care due to the COVID-19 epidemic. Nevertheless, even considering only HO-HCFA cases, the observed reduction was ~50%.

Despite the aforementioned limitations, our observation of a dramatic decrease in CDI in a context of no reduction in the use of antibiotics supports the importance of reducing the nosocomial transmission by healthcare workers or asymptomatically colonized patients, reinforcing cleaning procedure and reducing hospital mobility of patients in the epidemiological control of CDI.
