# Impact of the coronavirus disease 2019 (COVID-19) pandemic on nosocomial *Clostridioides difficile* infection — ERRATUM

**DOI:** 10.1017/ice.2020.1380

**Published:** 2021-02

**Authors:** Manuel Ponce-Alonso, Javier Sáez de la Fuente, Angela Rincón-Carlavilla, Paloma Moreno-Nunez, Laura Martínez-García, Rosa Escudero-Sánchez, Rosario Pintor, Sergio García-Fernández, Javier Cobo

**Affiliations:** 1 Servicio de Microbiología, Hospital Universitario Ramón y Cajal and Instituto Ramón y Cajal de Investigación Sanitaria, Madrid, Spain; 2 Red Española de Investigación en Patología Infecciosa, Madrid, Spain; 3 Servicio de Farmacia Hospitalaria, Hospital Universitario Ramón y Cajal and Instituto Ramón y Cajal de Investigación Sanitaria, Madrid, Spain; 4 Servicio de Medicina Preventiva, Hospital Universitario Ramón y Cajal and Instituto Ramón y Cajal de Investigación Sanitaria, Madrid, Spain; 5 Servicio de Enfermedades Infecciosas, Hospital Universitario Ramón y Cajal and Instituto Ramón y Cajal de Investigación Sanitaria, Madrid, Spain

In the above mentioned article by Ponce-Alonso et al^1^, the wrong figure file was used for Figure 1 in the final published version of record. The correct Figure 1 appears below. The publisher apologizes for the error.

**Fig. 1.**
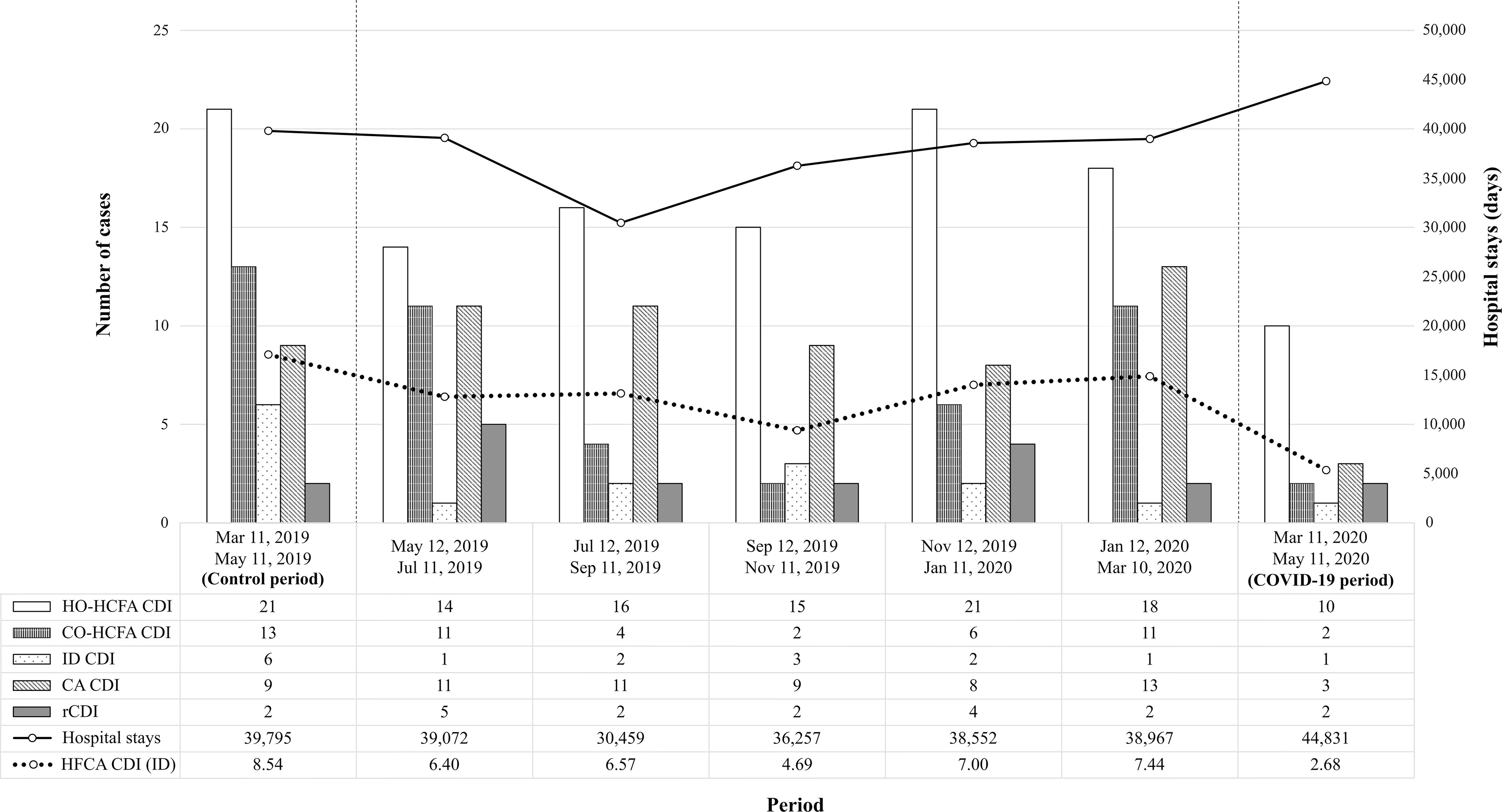
Evolution of *C. difficile* infection (CDI) over time, from control period (left) to COVID-19 period (right). The bar chart shows the total CDI case count, grouped by epidemiological definition. The solid line represents total hospital stays during each period (in days), which were used to calculate the incidence density of nosocomial CDI cases (dashed line). Note. HO-HCFA CDI, hospital-onset healthcare facility-associated *C. difficile* infection; CO-HCFA CDI, community-onset healthcare facility-associated *C. difficile* infection; ID CDI, indeterminate-onset *C. difficile* infection; CA CDI, community-acquired *C. difficile* infection; rCDI, recurrent *C. difficile* infection; HCFA CDI (ID), incidence density of healthcare facility-associated *C. difficile* infection.
